# Assessment of heavy metal contamination in fish, fruits, and vegetables in Southwest Nigeria: A systematic review

**DOI:** 10.12688/f1000research.157781.1

**Published:** 2024-11-26

**Authors:** Babafemi Laoye, Peter Olagbemide, Tolulope Ogunnusi, Oghenerobor Akpor

**Affiliations:** 1 Biological Sciences, Afe Babalola University, Ado Ekiti, Ekiti, Nigeria

**Keywords:** Fish, fruits, vegetables, PRISMA, Tilapia zilli, Chrysichthys nigrodigitatus, Clarias gariepinus, and Oreochromis niloticus

## Abstract

**Background:**

The aim of this systematic review was to investigate the prevalence of heavy metal contamination in fish, fruits, and vegetables in Southwest Nigeria. The review focused on studies published over a ten-year period, between 2014 and 2024.

**Methods:**

Articles used for the study were obtained by conducting a comprehensive literature search using several databases, including ResearchGate, Scopus, Google Scholar, ScienceDirect, and PubMed using Preferred Reporting Items for Systematic Reviews and Meta-Analyses (PRISMA). To identify relevant studies, a plethora of keywords were utilized to search for articles in the selected databases, including. Articles reporting heavy metal contamination in specified food products within the last decade were included.

**Results:**

Of the 10,212 initially identified articles, 64 met the inclusion criteria after thorough screening. The selected studies were predominantly conducted in Lagos (30), Ondo (8), and Ogun (7) states, with few studies in Oyo, Ekiti, and Osun states. The majority of the research focused on fish (40 studies), followed by vegetables (20) and fruits (4). The commonly studied fish species were observed to be *Tilapia zilli*, *Chrysichthys nigrodigitatus*, *Clarias gariepinus*, and *Oreochromis niloticus*, with heavy metal concentrations frequently exceeded WHO limits.

**Conclusions:**

Therefore, this review highlights the significant risks posed by the presence of heavy metals in food products and underscores the importance of stringent environmental monitoring and the adoption of appropriate regulatory mechanisms for health and environmental risk mitigation. This could help in the formulation of appropriate policy implementation strategies geared towards mitigating heavy metal contamination in the region’s food supply.

## 1. Introduction

Heavy metals cause build-ups and biological magnification in plethora food chains because they are sturdy and loiter in the ecosystem for a long period. In the Southwestern part of Nigeria, where industrialization, rapid urbanization, and agricultural activities greatly contribute to environmental pollution ( Odafivwotu and Abel, 2015; Akinnifesi *et al., *2021; Akinsola *et al., *2022) and stringent environmental regulations are seldom implemented, this presents a long-term debilitating health risk. The population is more dependent on locally produced food. Application of fertilizers, use of pesticides possessing heavy metal compounds, and use of unadulterated irrigation water can all contribute to heavy metal contamination in the soil, thereby affecting humans via consumption of agricultural produce. Fish in aquatic systems become contaminated by heavy metals in contaminated water bodies, frequently due to mining operations or industrial effluents. Although lead (Pb), cadmium (Cd), mercury (Hg), arsenic (As), aluminum (Al), and mercury (Hg) are not necessary for normal human functioning ( Boyd and Rajakaruna, 2013), food contamination by heavy metals poses a serious health risk to consumers, especially because these metals bio accumulate in the food chain ( Hussain *et al., *2019; Nkwunonwo *et al., *2020; Bawa-Allah 2023).

In Nigeria, fish, fruits, and vegetables contain dangerously high concentrations of heavy metals, often surpassing internationally acceptable limits set by regulatory bodies such as the Food and Agriculture Organization (FAO) and the World Health Organization (WHO). These findings have also been reported in other studies. Adegbola *et al*. (2021) and Abidemi-Iromini *et al.* (2022) conducted studies on fish from rivers in Lagos and Ogun states and revealed high concentrations of heavy metals, which are extremely harmful even in small amounts. Similarly, consuming grains (such as rice) and plants (including lettuce, amaranth, water spinach, and cowpea) cultivated in soil contaminated with mercury over an extended period of time is harmful to human health. Studies conducted in Southwest Nigeria have shown that commonly consumed food items contain elevated concentrations of heavy metals, frequently surpassing the permissible limits set by the Food and Agriculture Organization (FAO) and World Health Organization (WHO) ( Adekunle *et al., *2009; Akinola *et al., *2020). For example, fish samples from rivers near industrial zones in Lagos were found to contain large amounts of Pb and Cd by Olayinka and Adedeji (2018), whereas vegetables irrigated with contaminated water sources had high levels of arsenic ( Ogundele *et al., *2015).

Exposure to heavy metals is associated with numerous health hazards. Lead is a common heavy metal that contaminates the environment and enters the body through absorption, bioavailability, bioconcentration, and biomagnification. It causes problems in the reproductive, neurological, skeletal, hematological, renal, and cardiovascular systems ( Collin *et al., *2022). Mercury exposure causes neurological damage, including ataxia, muscle weakness, numb limbs, disturbance in speech, chewing, and swallowing, as well as brisk and increased tendon reflexes in patients exposed to massive amounts of Me-Hg ( Balali-Mood *et al., *2021). Cd exposure can result in a variety of adverse effects, including renal and hepatic dysfunction, pulmonary edema, testicular damage, osteomalacia, and damage to the adrenal and hematopoietic systems ( Genchi *et al., *2020). Long-term arsenic exposure can cause cancer, developmental problems, and skin damage. Additionally, long-term exposure to arsenic may suppress the immune system by causing keratinocyte apoptosis through the interaction of Fas and Fas-ligand, lowering the percentage of peripheral blood CD4+ T cells, reducing the number of Langerhans cells, and altering their migration ( Tang *et al., *2023). Understanding the distribution and concentrations of heavy metals in regularly consumed foods is essential for safeguarding public health in Southwest Nigeria, given their possible health consequences.

Furthermore, it is impossible to ignore how heavy metal poisoning affects society economically. Agriculture and fishing in Southwest Nigeria provide a substantial portion of the population’s income. These food supplies are contaminated, which puts the public’s health in danger, as well as the local economy, food security, and consumer confidence in the safety of locally made commodities at risk. This might result in more imported food products, which would drive up food prices and worsen poverty in areas that already have serious economic difficulties.

A rigorous study of the degree of heavy metal contamination is important in Southwest Nigeria because of the growing industrial and agricultural operations there, as well as possible health repercussions. The purpose of this systematic study was to evaluate the level of heavy metal contamination in Southwest Nigerian fish, fruits, and vegetables. This study reviews previous research in an effort to provide readers with a thorough understanding of contamination levels, pinpoint possible heavy metal sources, and assess the health hazards of consuming them. In addition, this study seeks to identify areas of unmet need in the field, indicate future research directions, and offer mitigation measures for heavy metal pollution in the area’s food supply. Designing policies and actions to lower public health risks, guarantee food safety, and advance environmental sustainability in Southwest Nigeria also require an understanding of the extent and effects of heavy metal contamination in food supplies.

## 2. Methods

### 2.1 Article search

For effective methodological integrity and validity, quality, PRISMA was utilized ( Haddaway *et al., *2022). For this review, a digital literature search was conducted to find articles between years 2014-2024 that as reported heavy metal contamination in food products in southwestern Nigeria. In this review, the research gate, scopus-indexed papers, connected papers, Google Scholar, Science Direct, and PubMed were the predominant databases employed. Each database search was carried out independently, and the selected articles were juxtaposed together. For this review, a plethora of keywords were utilized to search for articles on the selected databases, including heavy metal contamination, pollution, toxic metals, lead, cadmium, cobalt, mercury, arsenic, chromium, manganese, nickel, cadmium, fruits, vegetables, fish, Southwest Nigeria, Nigeria, health risks, toxicity, exposure assessment, bioaccumulation, cross-sectional study, case study, environmental monitoring, and survey. Furthermore, Boolean operators (AND and OR) was combined with “heavy metals’ which is the predominant key word to search for articles more effectively.

### 2.2 Inclusion and exclusion criteria

The inclusion criteria were articles published within the last 10 years (2014-2024), that reported heavy metal contamination in fish, fruits, and vegetables in southwest Nigeria. Articles that did not report heavy metal contamination in fish, fruits, or vegetables were excluded from this study.

### 2.3 Articles selection

A custom range between the years 2014-2024 was used to select articles that reported contamination of heavy metals in fish, fruits, and vegetables in the last 10 years. Thorough scrutiny of all articles by reading the titles and abstracts was performed to select the preferred articles for this study. A total of 61 articles that reported heavy metal contamination in fish, fruits, and vegetables in Southwest Nigeria were selected for this study. Overall, 10, 212 articles were obtained from different databases. Specifically, Google Scholar yielded 8,420 hits, PubMed yielded 58 hits, Research Gate yielded 1,487 hits, and Scopus yielded 247 hits. During this review, 850 articles were removed before screening leading 9,362 articles left. Critical reading was performed to select non-eligible articles with 9, 362 articles being screened. During this review, 8,538 articles were further excluded after custom screening. Furthermore, 824 articles were sought for retrieval, and 4 articles were further removed (820 articles), 756 inconsequential articles were excluded to avoid meeting the inclusion criteria. Specifically, 44 articles were deleted as the research methodology was not well defined; 210 articles were omitted because they fell outside the 10 years ago criteria; 5 articles were removed because they were not peer reviewed; 240 articles were deleted as they did not focus on heavy metal contamination in either fish, vegetables, or fruits; 8 articles were deleted as they were books and reviews; and 313 articles were excluded because the research was not domiciled in the southwestern region of Nigeria. A total of 64 articles were included in this review.

## 3. Results

Among the 64 eligible articles selected for this systematic review, 30 were carried out in Lagos, eight in Ondo, and seven in Ogun State of Nigeria. Three, six, and seven studies were conducted in the Oyo, Ekiti, and Osun states, respectively. The majority (40) of the studies included in this review were carried out on fish, while 20 and 4 studies were carried out on vegetables and fruits, respectively

### 3.1 Heavy metal contamination in Fish

In the studies reviewed, a plethora of fish species, particularly *Tilapia zilli*, *Chrysichthys nigrodigitatus*, *Clarias gariepinus*, *Oreochromis niloticus*, frequently exceeded the World Health Organization (WHO) limits for heavy metals, such as lead (Pb), chromium (Cr), and Nickel (Ni.) Multiple studies have reviewed the constant pollution of water bodies where these fish species are found, which poses health risks to humans via consumption. *Tilapia zilli* and *Chrysichthys nigrodigitatus* were found to be highly prone to pollution and contamination by heavy metals, often exceeding the WHO permissible limits for zinc (Zn), Cr, and Pb. In addition, *Clarias gariepinus* showed significant contamination in some articles, with remarkably elevated levels of cadmium (Cd) and lead (Pb). *Oreochromis niloticus* (Nile tilapia) typically showed lower contamination levels, except for lead (Pb) in some studies. Heavy metal pollution in staple foods such as fish, fruits, and vegetables is of great concern and cannot be overemphasized. In this review, lead (Pb) was mostly reported to be above safe consumption levels in many fish samples, which was reflected in studies by Yahaya *et al.* (2022) and Olujimi *et al.* (2017), which showed particularly high concentrations. Chromium (Cr) and nickel (Ni) are frequently found above safe limits, particularly in studies by Yahaya *et al.* (2022) and Balogun *et al.* (2021).

In addition, Zinc (Zn) and Iron (Fe) were found in some fish samples, exceeding the WHO limits in studies by Ajibare *et al.* (2021). In the articles reviewed in this study, *Clarias gariepinus* and *Sarotherodon melanotheron* were low or within the WHO safe limits of contamination in articles reviewed in this study ( Adetutu *et al., *2023; Akinjogunla *et al., *2023). Shell fish species, such as *Callinectes danae* and *Cardisoma armatum* showed copper contamination, exceeding the WHO limits, as reported by Oladunjoye *et al.* (2021).

From 2014 to 2024, heavy metal contamination patterns reflect persistence, revealing continuous environmental pollution issues. Consumption of fish contaminated with heavy metals poses a significant risk to human health, which may result in kidney diseases and neurological damage *. Clarias gariepinus* was reflected in a plethora of articles used for this systematic review, possibly because of its commercial importance. The less frequently detected metals in fish samples in this systematic review were mercury (Hg) and arsenic (As), as they were not tested using an Atomic Absorption Spectrophotometer (AAS), their levels were below detection limits (BDL), or were generally not detected (ND), indicating either minimal contamination [Table 1 Extended data. Akpor *et al.* (2024)].

### 3.2 Heavy metal contamination in vegetables

The results of this systematic review revealed that a plethora of vegetables contained heavy metals that exceeded the WHO permissible limits, connoting potential health risks to humans via consumption. High levels of cadmium (3.52 mg/kg) chromium (49.13 mg/kg) and lead (9.97 mg/kg) were detected in *Talinum triangulare* in studies conducted by Atikpo *et al., *2021, indicating the vegetable accumulate multiple heavy metals which is very much dangerous to the health of humans. Green peas ( *Pisum sativum*) have elevated levels of Pb and Al ( Ojezele *et al., *2021). These heavy metal levels in *Pisum sativum* in this study transcend the WHO safe limits, indicating that consumption of peas is potentially dangerous for consumption ( Ojezele *et al., *2021). *Celosia argentea*, commonly known as plumed cockscomb red spinach, efo shoko, and spinach, repeatedly showed high levels of heavy metals in different studies. For example, Omoboyowa *et al.* (2019) recorded a high zinc (Zn) concentration of 52.00 mg/kg, well above WHO limits. In another study, Agboola *et al.* (2023) found Cr at 3.90 mg/kg and Zn 7.30 mg/kg, both exceeding the threshold, indicating remarkable contamination. *Jatropha podagrica* commonly called botuje in southwest Nigeria, has heavy metals such as copper (0.210 mg/kg) and zinc (0.220 mg/kg), which are above the WHO limits, also suggesting a hazardous effect on human health ( Ogunwale *et al., *2021). In addition to elevated concentrations of heavy metals, this systematic review of vegetables also revealed heavy metal contamination within safety limits in a number of vegetables such as tomato, red pepper, bell pepper, chilli pepper, and red pepper. Similarly, all vegetables assayed by Farombi *et al.* (2020) were within the WHO safety levels (Cd, Cr, Co, Pb, Zn, and Ni) [Table 2 Extended data. Akpor *et al.* (2024)].

Moreso, an article by Ofudje *et al.* (2017), revealed acceptable levels of Zn and Mn in *Telfairia occidentalis,* suggesting regional differences in contamination. Among all vegetables in this review, cadmium and lead were revealed to be the prominent elevated metals in terms of concentration that appeared in this study. *Amaranthus hybridus, Corchorus olitorius, and Celosia argentea* revealed lead contamination in this study, and a build-up of lead in the body via consumption may lead to neurological disorders. Furthermore, heavy metals, such as Zn and Cu, were often found among vegetables in this review, exceeding the WHO limits, which was reflected precisely in *Celosia argentea* and *Jatropha podagrica.* Ojezele *et al.* (2021) reported elevated aluminum concentrations in green peas and mushrooms. (Table 2).

### 3.3 Heavy metal contamination in fruits

The results of this systematic review revealed only four articles that reported heavy metal contamination in southwest Nigeria; cadmium levels were reported in *Carica papya* at a concentration of 0.077 mg/kg, while Pb levels reached 0.074 mg/kg, with both heavy metals exceeding the WHO safety limits ( Ogunkunle *et al., *2014). Similarly, cobalt, manganese, and copper were also detected, but within permissible limits. Prolonged consumption of papaya from this area may pose a potential health risk. For *Citrullus lanatus* (watermelon), lead contamination was elevated at 1.760 mg/kg, which is well above the permissible limit, making it a cause for concern. Similarly, an article by Oladele and Aladesanmi (2020) also reported heavy metal contamination, but precisely in chromium and nickel, slightly exceeding the WHO safe limits. Results from this systematic review revealed lead contamination in *Mangifera indica* (Mango), which was recorded at 1.620 mg/kg, significantly exceeding the safety limit. Cadmium (0.091 mg/kg) and cobalt (0.029 mg/kg) were also detected, with Cd levels advancing towards the permissible limit, which raises concern over potential long-term health impacts from consuming contaminated mangoes ( Ogunkunle *et al., *2014). *Malus domestica* (apple) also revealed heavy metal contamination exceeding safe limits: chromium (0.300 mg/kg) and nickel (0.475 mg/kg), both of which are significantly above the WHO limits. ( Omoyajowo *et al., *2017). Furthermore, mixed Fruits were also reported to be contaminated in heavy metals: Lead, nickel and aluminum were found at 1.40 mg/kg, 0.625 mg/kg, and 7.00 mg/kg, respectively, with all exceeding permissible limits. In addition, orange ( *Citrus sinensis)* in an article by Omoyajowo *et al.* (2017) showed safe levels of heavy metal contamination, chromium (0.080 mg/kg) and lead (0.120 mg/kg), both within the WHO guidelines [Table 3 Extended data. Akpor *et al.* (2024)].

## 4. Discussion

The results of this review revealed widespread contamination of fruits, vegetables, and fish by heavy metals across southwestern Nigeria, with no significant public health concerns. Heavy metals, such as lead (Pb), cadmium (Cd), chromium (Cr), nickel (Ni), and zinc (Zn), were found to exceed the WHO safety limits for fish, fruits, and vegetables. These findings raise global concerns regarding the persistence of heavy metals in the environment due to pollution, industrial activities, and agricultural practices. In addition, widespread heavy metal contamination across different types of fruits in this systematic review study was highly reflected in this study, posing debilitating health risks, for example, from consuming fruits ( Ojezele *et al., *2021). Nonetheless, some articles have revealed fruits with heavy metal contamination within the WHO safe limits. *Citrullus lanatus* (Watermelon) displays safe levels of heavy metals, including cadmium (0.004 mg/kg) and lead ( Atikpo *et al., *2021).

Additionally, fish species, distinctly *Tilapia zilli, Chrysichthys nigrodigitatus,* and *Clarias gariepinus*, frequently exceeded the WHO limits for heavy metals, particularly Pb, Cr, and Ni. Results from this study in terms fish contamination in an article by Yahaya *et al.* (2020) revealed lead, and chromium contamination at levels exceeding WHO linits in *Tilapia zilli* with 4.36 mg/kg and 5.40 mg/kg respectively. This was similar to the results from a study by Putshaka *et al.* (2015), who reported contamination levels in lead and chromium at 1.132 mg/kg and 1.845, respectively, exceeding. The Pb level in the study by Yahaya *et al.* (2020) was much higher than that reported by Putshaka *et al.* (2015). The results of this study by Yahaya *et al.* (2020) were dissimilar to results from a study by Inalegwu *et al.* (2019), who reported lower levels of Pb (0.347 mg/kg) and Cr (0.848 mg/kg) contamination in *Tilapia zilli* when compared to the study by Yahaya *et al.* (2020). This suggests that the aquatic environments in which these fish are harvested are heavily contaminated, most likely from industrial runoff, mining activities, and illegitimate waste disposal. This raises public health concerns, as fish are a primary source of dietary protein globally, and long-term consumption of polluted or contaminated fish may lead to bioaccumulation of toxic metals in humans, resulting in kidney damage, congenital defects, and an increased risk of cancer.

Yahaya *et al.* (2020) reported Pb, Cu, and Ni contamination in *Chrysichthys nigrodigitatus* exceeding the WHO limits of 3.63 mg/kg, 1.28 mg/kg and 4.16 mg/kg respectively. The results from Yahaya *et al.* (2020) were similar to the results from Unaeze *et al*. (2023). Although, the level of Pb contamination was lower (2.00 mg/kg) in the study by Unaeze *et al.* (2023) when compared to the study by Yahaya *et al.* (2020) but exceeding WHO limit, also the concentration for Zinc in *Chrysichthys nigrodigitatus* was extremely high (240.00 mg/kg) in the study by Unaeze *et al.* (2023) unlike results from Yahaya *et al.* (2020) having Zinc level in the same fish as 1.28 mg/kg. Also Cu levels in Unaeze *et al.* (2023) (2.32 mg/kg) was higher than the level reported by Yahaya *et al.* 2020 (1.28 mg/kg) with both exceeding WHO limits.

*Clarias gariepinus* is a commercially liked fish species, which was reflected several times in the reviewed articles selected for this study, probably because of its significance in the local markets in Nigeria. The high levels of Cd and Pb found in this species underscore the need for stringent monitoring of fish harvested from polluted water bodies. In this review article, seven articles revealed heavy metal contamination in *Clarias gariepinus* ( Olurin, 2024; Ogungbemi *et al., *2022; Utete and Fregene, 2020; Yahaya *et al., *2022; Ayanda *et al., *2020; Adubiaro and Animashaun, 2021, Adewunmi *et al., *2017), while, four articles ( Olurin, 2024; Utete and Fregene, 2020; Yahaya *et al., *2022; Ayanda *et al., *2020) reported heavy metal contamination exceeding WHO limits, and three articles ( Ogungbemi *et al., *2022; Adubiaro and Animashaun, 2021; Adewunmi *et al., *2017) reported heavy metal contamination within the WHO permissible limits. In this study, heavy metal contamination in *Clarias gariepinus* exceeding WHO limits is similar to the study by Odey *et al.* (2020), with Lead (Pb) being at the forefront, frequently appearing in these articles. Articles from this review reporting Pb contamination revealed Pb levels at levels of 9.14 mg/kg, 0.20 mg/kg, 3.23 mg/kg, 9.43 mg/kg, 11.10 mg/kg, 92.08 mg/kg, and 1.10 mg/kg in studies by Yahaya *et al. *(2022), Ogungbemi *et al. *(2022), Olurin (2024), Adewunmi *et al. *(2017), Utete and Fregene (2020), Ayanda *et al. *(2020) and Adubiaro and Animashaun (2021) respectively. Pb level (92.08 mg/kg) reported in *Clarias gariepinus* reported by Ayanda *et al.* (2020) was higher than that reported by Odey *et al.(*2020), although they all exceeded WHO permissible limits. However the Pb level reported by Olurin (2024) was lower than what was reported by Odey *et al.* (2020) More so, articles by Adubiaro and Animashaun (2021), Ogungbemi *et al. *(2022), Adewunmi *et al.* (2017) reported heavy metal contamination within WHO permissible limits in all selected metals that was assayed for, results in these studies were similar to results reported by Abubakar and Adeshına (2019). The presence of Pb in the main culprits of this fish implies that ingestion of *Clarias gariepinus* may be poisonous to individuals in the southwest region of Nigeria, as long-term ingestion of Pb via this fish leads to the accumulation of Pb, which may result in coma, convulsions, and even death ( Ray & Vashishth, 2024).

Furthermore, some articles reviewed in this study found metals such as zinc and iron exceeding WHO safety limits ( Ajibare *et al., *2021; Oghenechuko *et al., *2022; Taiwo *et al., *2019; Wangboje & Miller 2018; Taiwo *et al., *2017; Taiwo *et al., *2019; Olowu *et al., *2015) Articles that reported heavy Zn conatamination in fish were similar to results reported by Mahidi and Albashr (2023); Yalwa *et al. *(2024) and Balooch *et al. *(2021) in terms of exceeding WHO limits Furthermore, Ajibare *et al.* (2021) reported Znc (112.19 mg/kg) contamination in *Oreochromis* which was higher than Znc level reported Mahidi and Albashr (2023); Yalwa *et al. *(2024) and Balooch *et al. *(2021) in fish at a level of 37.17 mg/kg, 50.84 mg/kg and 13.36 mh/kg respectively. Bioaccumulation of Zn may cause debilitating effects in the human body, resulting in reproductive disorders and Alzheimer’s disease ( Kiouri *et al., *2023) Although, Fe is an essential element for humans as it participates in a wide variety of metabolic processes, including oxygen transport, deoxyribonucleic acid (DNA) synthesis, and electron transport ( Abbaspour *et al., *2014). For Fe contamination of fish, reports from studies by Taiwo *et al.* (2017); Olowu *et al. *(2015); Taiwo *et al. *(2019) and Oghenechuko *et al. *(2022) revealed higher level of Fe concentration in fish at level of 8.05 mg/kg/, 8.02 mg/kg, 8.68 mg/kg and 5.51 mg/kg respectively when compared to Fe levels that was reported in studies by Bobar *et al.* (2022) and Salam *et al.* (2019) having with 0.23 mg/kg and 4.30 mg/kg respectively; suggesting a great concern in the southwestern region in Nigeria, as bioaccumulation of Fe. The ingestion of fish by humans may lead to the formation of free radicals and excessive tissue damage in the body ( Abbaspour *et al., *2014). Shellfish species *like Callinectes danae* and *Cardisoma armatum* also revealed levels of copper contamination, indicating that both fish and crustaceans from these regions may pose a health risk if consumed in large quantities ( Oladunjoye *et al., *2021). Because of the continual nature of heavy metals in aquatic habitats, the continuous consumption of contaminated fish could have long-term health implications for the southwestern people of Nigeria.

Vegetables and fruits are very much essential components of human diets ( Rodriguez-Casado, 2016) Results from this review showed substantial contamination with heavy metals, particularly lead and cadmium. *Carica papaya* (pawpaw) and *Mangifera indica* (mango), the two frequently reported fruits captured in this systematic review, were found to contain elevated levels of lead, significantly exceeding the WHO limits. Imperatively, Pb levels in mangoes reach 1.620 mg/kg, posing a serious health risk, as chronic exposure to Pb can lead to neurological disorders, developmental delays in children, and cardiovascular diseases in adults ( Atikpo *et al., *2021). In the same fashion, levels in pawpaw (0.077 mg/kg) approached the safety threshold, raising concerns over kidney damage and bone demineralization with long-term consumption leading to bioaccumulation. Watermelon ( *Citrullus lanatus*) also exhibited notable heavy metal contamination, with Pb levels reaching 1.760 mg/kg, which is significantly higher than the permissible limits. This finding is concerning given the popularity of watermelon in Southwestern Nigeria, suggesting that regular consumption could lead to bioaccumulation of heavy metals in humans, who are invariably consumers. While some fruits such as apples ( *Malus domestica*) and mixed fruit samples showed contamination with metals such as chromium and nickel, other fruits such as oranges ( *Citrus sinensis*) revealed metal levels exceeding WHO safe limits ( Omoyajowo *et al., *2017).

Similarly, vegetables, a very important food in the diets of individuals in southwestern Nigeria, revealed alarming levels of contamination. *Talinum triangulare*, green peas ( *Pisum sativum*), and *Celosia argentea were* reported to contain high concentrations of Cd, Cr, Zn, and Pb. The presence of a plethora of heavy metals in these vegetables, most importantly lead and cadmium, is also of great concern, as these metals can cause cumulative dangerous effects over time, leading to severe health issues, including neurotoxicity, reproductive issues, and carcinogenic effects ( Rodriguez-Casado, 2016).

The high levels of aluminum in green peas and mushrooms in this systematic review ( Ojezele *et al., *2021) further suggest the potential for widespread heavy metal contamination, as aluminum has been implicated in anemia and neurological disorders ( Cirovic *et al., *2023). In addition to aluminum, lead was also reported in a study by Ojezele *et al*. (2021), which contaminates green peas and mushrooms at high levels. Precisely, for green peas Al (14.0 mg/kg), Pb (1.68 mg/kg), and for mushroom Al (11.0 mg/kg) Pb (1.38 mg/kg). The results from the study by Ojezele *et al.* (2021) were dissimilar to those of Pb levels reported by Oloruntoba *et al.* (2017), which were below detectable limits. For mushroom, the Pb level(1.15 mg/kg) reported by Orywal *et al.* (2021) was slightly lower than the Pb (1.38 mg/kg) level in mushroom reported by Ojezele *et al.* (2021), with mushrooms being of great concern in the southwest region of Nigeria. The accumulation of aluminum in vegetables may lead to anemia and neurological disorders, making it a metal of great concern. Overall, *Amaranthus hybridus* (Pb and Cd), *Telfairia occidentalis* (Cu and Pb), and *Celosia argentea* (Cr, Zn, and Cu) had heavy metal concentrations exceeding the WHO limits, having a robust tendency to cause debilitating effects on human health. Furthermore, some vegetables, such as red peppers, chilli peppers, and tomatoes, were found to have heavy metal levels according to the WHO safety guidelines, suggesting possible regional variations in contamination. The remarkable levels of contamination among various food groups indicate that stringent measures by the government, private organizations, and environmental management strategies must be strengthened to reduce these risks. This review highlights the persistent contamination of essential food items in Southwest Nigeria, which may have profound long-term health consequences. Chronic exposure to elevated levels of lead, cadmium, and other heavy metals is linked to a range of adverse health effects, including neurotoxicity ( Mitra *et al., *2022) cardiovascular diseases, and increased cancer risks ( Ebrahimi, *et al., *2020) Lead exposure, particularly in children, can cause irreversible cognitive impairments, whereas cadmium accumulation in the body is associated with kidney dysfunction and osteoporosis ( Ma *et al., *2022).

## 5. Conclusion

This systematic review highlights the prevalence and persistence of heavy metal contamination in fish, fruits, and vegetables in Southwest Nigeria between 2014 and 2024. Most of the reviewed studies revealed significant contamination in fish, fruits, and vegetables. The reviewed studies revealed that the concentrations of chromium and nickel frequently exceeded the permissible limits of the World Health Organization (WHO), which could pose significant health risks to consumers. A number of fish species ( *Tilapia zilli*, *Chrysichthys nigrodigitatus*, and *Clarias gariepinus*) in the reviewed studies, showed high levels of contamination, thus making them critical indicators of environmental pollution in the region. Although vegetables and fruits have been less frequently studied in the region, the reviewed studies showed that they still exhibited significant levels of heavy metal contamination in some instances. Therefore, this review highlights the significant risks posed by the presence of heavy metals in food products and underscores the importance of stringent environmental monitoring and the adoption of appropriate regulatory mechanisms for health and environmental risk mitigation. This could help in the formulation of appropriate policy implementation strategies geared towards mitigating heavy metal contamination in the region’s food supply.

## Ethics and consent

Ethical approval and consent were not required.

## Data Availability

No data are associated with this article. Figshare repository - “Manuscript Tables, PRISMA flowchart and PRISMA Checklist for Systematic Review on Heavy Metal Contamination of Fish, Fruits and Vegetables in Southwest, Nigeria”. https://doi.org/10.6084/m9.figshare.27453258.v3 ( Akpor *et al.*, 2024). This project contains following datasets:
1.
Table 1_Levels of Heavy Metal Contamination in Various Fish in Southwest Nigeria.docx 2.
Table 2_Levels of Heavy Metal Contamination in Various Vegetables in Southwest Nigeria.docx 3.
Table 3_Levels of Heavy Metal Contamination in Various Fruits in Southwest Nigeria.docx 4. PRISMA flow chart systematic review.docx 5. PRISMA abstract Checklist.docx 6. PRISMA Main text checklist 7. Systematic review Table references Data are available under the terms of the Creative Commons Zero “No rights reserved” data waiver (CC0 1.0 Public domain dedication). Figshare repository - PRISMA checklist for ‘Assessment of Heavy Metal Contamination in Fish, Fruits and Vegetables in Southwest Nigeria: A Systematic Review’. https://doi.org/10.6084/m9.figshare.27453258.v3 ( Akpor *et al.*, 2024). Data are available under the terms of the Creative Commons Zero “No rights reserved” data waiver (CC0 1.0 Public domain dedication).
