# Supplementary material for: Assessment of heavy metal contamination in fish, fruits, and vegetables in Southwest Nigeria: A systematic review
Source: F1000Res. 2025 Jan 23;13:1430. Originally published 2024 Nov 26. [Version 2] doi: 10.12688/f1000research.157781.2 (PMC12375182; doi:10.12688/f1000research.157781.2)
Supplement: Peer review history

# Reviewer report: Reviewer response for version 2

**DOI:** 10.5256/f1000research.177164.r406133

**Published:** 2025-08-23

**Authors:** Mathias Nzitiri Bwala

**Affiliations:** 1 National Environmental Standards and Regulations Enforcement Agency, Maiduguri, Nigeria

The manuscript is okay in its present form as most of the recommendations by the previous reviewers were adequately taken care of.

 In the abstract (under the methods sub-section), the second sentence "To identify relevant studies, a plethora of keywords were utilized to search for articles in the selected databases, ***including*** ". the word "including" there should be removed.

 Overall, the review is okay.

 Thank you,

 Bwala M.N

Are the rationale for, and objectives of, the Systematic Review clearly stated?

Yes

Is the statistical analysis and its interpretation appropriate?

Yes

If this is a Living Systematic Review, is the ‘living’ method appropriate and is the search schedule clearly defined and justified? (‘Living Systematic Review’ or a variation of this term should be included in the title.)

Not applicable

Are sufficient details of the methods and analysis provided to allow replication by others?

Yes

Are the conclusions drawn adequately supported by the results presented in the review?

Yes

Reviewer Expertise:

Environmental Biology; Ecotoxicology; Ecology

I confirm that I have read this submission and believe that I have an appropriate level of expertise to confirm that it is of an acceptable scientific standard.

# Reviewer report: Reviewer response for version 2

**DOI:** 10.5256/f1000research.177164.r362052

**Published:** 2025-02-14

**Authors:** Harry R. Yucra-Condori, Sheyla J Angulo Vargas

**Affiliations:** 1 Academic Department of Food Industries Engineering, National University of Saint Augustin of Arequipa, Arequipa, Arequipa, Peru; 2 National University of San Agustin, Arequipa, Arequipa, Peru

After a comprehensive analysis of the attached document, various improvements implemented by the authors in response to the reviewers' observations have been identified, as well as some deficiencies that still require attention. A detailed summary of these findings is presented below:

 Significant Improvements

1. Expanded justification for the 2014-2024 study period (page 3).
2. Improved description of the article selection process, including details on the number of reviewers and the stages of the process (pages 4-5).
3. Inclusion of study limitations in the conclusion (page 10).
4. Clearer and more structured presentation of results (pages 5-10).
5. Partial standardization of data presented in the tables (pages 19-34).

Persistent Deficiencies

1. Inconsistency in the presentation of results on heavy metal contamination in fish, limited to indicating whether WHO limits are exceeded or not, in contrast to other sections that offer numerical values.
2. Lack of uniformity in the presentation of data in the tables, specifically:
  - Absence of units in Tables 1 and 2.
  - Use of inconsistent and potentially confusing terms (e.g., "Yes", "No", "exceeded limit", "All within WHO limits except...", etc.).
3. Inconsistency in the use of the abbreviation "ND", which in some tables means "not determined" and in others "not detected".

Conclusion

 The authors have made notable improvements in response to the initial observations. However, aspects that require additional revision persist to ensure the coherence, clarity, and rigor of the study. It is recommended to address the mentioned deficiencies to elevate the overall quality of the document and facilitate its understanding by readers.

Are the rationale for, and objectives of, the Systematic Review clearly stated?

Partly

Is the statistical analysis and its interpretation appropriate?

Not applicable

If this is a Living Systematic Review, is the ‘living’ method appropriate and is the search schedule clearly defined and justified? (‘Living Systematic Review’ or a variation of this term should be included in the title.)

Not applicable

Are sufficient details of the methods and analysis provided to allow replication by others?

Partly

Are the conclusions drawn adequately supported by the results presented in the review?

Partly

Reviewer Expertise:

Environmental remediation, environmental contamination and health risk, food design.

We confirm that we have read this submission and believe that we have an appropriate level of expertise to confirm that it is of an acceptable scientific standard.

# Reviewer report: Reviewer response for version 1

**DOI:** 10.5256/f1000research.173282.r354120

**Published:** 2025-01-13

**Authors:** Harry R. Yucra-Condori, Sheyla J Angulo Vargas

**Affiliations:** 1 Academic Department of Food Industries Engineering, National University of Saint Augustin of Arequipa, Arequipa, Arequipa, Peru; 2 National University of San Agustin, Arequipa, Arequipa, Peru

**Report**

 Systematic Review of Heavy Metal Contamination in Fish, Fruits, and Vegetables in Southwestern Nigeria (2014-2024)

**Abstract**

 This comprehensive systematic review meticulously examines heavy metal contamination in fish, fruits, and vegetables in southwestern Nigeria over the decade spanning 2014-2024. The researchers conducted an exhaustive search across multiple scientific databases, rigorously selecting 64 studies that met specific inclusion criteria.

 Key Findings

 The analysis reveals alarming levels of contamination, particularly by lead, cadmium, chromium, and zinc, in various fish species and some vegetables, frequently exceeding the limits established by the World Health Organization (WHO). Although less extensively studied, fruits also exhibited a degree of contamination.

 Methodology Enhancement Recommendations

 Research Foundation and Objectives

- Formulate specific objectives and clear research questions.
- Provide a detailed justification for the selection of the 2014-2024 study period.

Methodological Approach and Data Analysis

- Delineate the complete search strategy for at least one database.
- Specify the study selection process, including the number of reviewers at each stage.
- Describe data extraction and synthesis methodologies.
- Incorporate a quality assessment or risk of bias evaluation for included studies.

Conclusion Formulation

- Explicitly link conclusions to the presented data.
- Discuss the quality and consistency of the evidence.
- Address limitations of both included studies and the review itself.

Additional Considerations

1. **Health Risk Assessment**: Conduct a more rigorous analysis considering exposure levels and consumption patterns, utilizing guidance from articles such as:
  - Román-Ochoa Y, et al., 2021 (Ref 1)
  - Choquenaira-Quispe C, et al., 2022 (Ref 2)
2. **Inter-Study Variability**: Discuss potential reasons for observed discrepancies.
3. **Practical Implications**: Address consequences for consumers and public health authorities.
4. **Sensitivity Analysis**: Consider including an assessment of evidence certainty (e.g., using GRADE methodology).
5. **Keywords**: Review relevance to facilitate web-based identification of the review.
6. **Figure 1**: Include a brief justification for selection criteria and the number of participating reviewers.
7. **Cereal Inclusion**: Justify the exclusion of cereals, considering their importance in local diets as evidenced by studies such as:
  - Sowunmi F, et al., 2020 (Ref 3)
  - Ikudayisi A, 2020 (Ref 4)
  - Otemuyiwa IO, et al., 2012 (Ref 5)
8. **Results Presentation Uniformity**: Standardize the presentation of concentration data across all sections (fish, vegetables, and fruits).
9. **Table Structure**: Uniformize format, including units, complete row numbering, WHO maximum values, and justify the absence of references in some rows.
10. **Legends and Abbreviations**: Include explanations for BDL, ND, and clarify terms such as "Si" and "exceeds limit" in the WHO limits column.
11. **Geographic Information**: Add a column in tables identifying the specific study region.
12. **Data Format**: Standardize the number of decimal places and metal nomenclature (acronyms or full names).
13. **Article Accessibility**: Identify the quantity of open access and subscription articles utilized.
14. It is suggested to provide quantitative summaries of contamination levels by food type and metal (ranges, medians).

Are the rationale for, and objectives of, the Systematic Review clearly stated?

Partly

Is the statistical analysis and its interpretation appropriate?

Not applicable

If this is a Living Systematic Review, is the ‘living’ method appropriate and is the search schedule clearly defined and justified? (‘Living Systematic Review’ or a variation of this term should be included in the title.)

Not applicable

Are sufficient details of the methods and analysis provided to allow replication by others?

Partly

Are the conclusions drawn adequately supported by the results presented in the review?

Partly

Reviewer Expertise:

Environmental remediation, environmental contamination and health risk, food design.

We confirm that we have read this submission and believe that we have an appropriate level of expertise to confirm that it is of an acceptable scientific standard, however we have significant reservations, as outlined above.

## References

[1] : Heavy metal contamination and health risk assessment in grains and grain-based processed food in Arequipa region of Peru. *Chemosphere* .2021;274: 10.1016/j.chemosphere.2021.129792 129792 10.1016/j.chemosphere.2021.129792 33556663

[2] : Quantification and health risk assessment of lead and cadmium in wheat, rice, and their processed products from Peru. *J Environ Sci Health B* .2022;57(4) : 10.1080/03601234.2022.2049152 297-304 10.1080/03601234.2022.2049152 35277121

[3] : Household demand for basic foodstuffs in a recessed economy: a case study of Southwest Nigeria. *World Journal of Science, Technology and Sustainable Development* .2020;17(1) : 10.1108/WJSTSD-06-2019-0029 1-19 10.1108/WJSTSD-06-2019-0029

[4] : Exploring Urban Dietary Pattern in Nigerian Households: A Case for Nutrient Adequacy. *Asian Journal of Advanced Research and Reports* .2020; 10.9734/ajarr/2020/v13i430315 24-34 10.9734/ajarr/2020/v13i430315

[5] : Food choice and meal consumption pattern among undergraduate students in two universities in Southwestern Nigeria. *Nutr Health* .2012;21(4) : 10.1177/0260106013510994 233-45 10.1177/0260106013510994 24620005

# Response

**Published:** 2025-01-17

**Authors:** Babafemi Laoye

**Affiliations:** Biological Sciences, Afe Babalola University, Ado Ekiti, Ekiti, Nigeria

Dear Reviewer,

 Thank you so much for the review comments. The authors are grateful for the comments. Below are responses to the review:

**Reviewer’s comment:** Methodology Enhancement Recommendations

 Research Foundation and Objectives

- Formulate specific objectives and clear research questions.
- Provide a detailed justification for the selection of the 2014-2024 study period.

Methodological Approach and Data Analysis

- Delineate the complete search strategy for at least one database.
- Specify the study selection process, including the number of reviewers at each stage.
- Describe data extraction and synthesis methodologies.

Incorporate a quality assessment or risk of bias evaluation for included studies.

**Authors’ response: **This has been attended to as revised according. Justification for selection of study period included

**Reviewer’s comment: **Conclusion Formulation

- Explicitly link conclusions to the presented data.
- Discuss the quality and consistency of the evidence.
- Address limitations of both included studies and the review itself.

**Authors’ response: **The conclusion has been revised and limitation included

**Reviewer’s comment: **Additional Considerations

1. **Health Risk Assessment**: Conduct a more rigorous analysis considering exposure levels and consumption patterns, utilizing guidance from articles such as:
  - Román-Ochoa Y, et al., 2021 (Ref 1)

Choquenaira-Quispe C, et al., 2022 (Ref 2)

**Authors’ response: **Health risk assessment was not part of the study objectives. The study was a systematic review, so the authors had no access to data on exposure and consumption levels of the food items. If it was an original research article, the authors would have considered that.

**Reviewer’s comment: Inter-study variability**: Discuss potential reasons for observed discrepancies.

**Authors’ response: **This has been indicated in the limitation.

**Reviewer’s comment: Practical Implications**: Address consequences for consumers and public health authorities.

**Authors’ response: **This is reflected in the discussion

**Reviewer’s comment: Sensitivity Analysis**: Consider including an assessment of evidence certainty (e.g., using GRADE methodology).

**Authors’ response: **The PRIMA protocol was holistically followed during the course of the study

**Reviewer’s comment: Keywords**: Review relevance to facilitate web-based identification of the review.

**Authors’ response: **Keywords have been added

**Reviewer’s comment: Figure 1**: Include a brief justification for selection criteria and the number of participating reviewers.

**Authors’ response: **This is already reflected in the article selection section

**Reviewer’s comment: Cereal Inclusion**: Justify the exclusion of cereals, considering their importance in local diets as evidenced by studies such as:

-
  - Sowunmi F, et al., 2020 (Ref 3)
  - Ikudayisi A, 2020 (Ref 4)

Otemuyiwa IO, et al., 2012 (Ref 5)

**Authors’ response: The inclusion** of cereal was outside the objectives of the study. The study was solely focused on fish, fruits, and vegetables. There is no way all food products can be included in a single study. The authors may consider that in subsequent studies.

**Reviewer’s comment: Results Presentation Uniformity**: Standardize the presentation of concentration data across all sections (fish, vegetables, and fruits).

**Authors’ response: **Although that would have been the ideal thing to do, data were extracted from the information provided by the authors of the manuscript. We have no option but to report exactly what they provided and make our interpretation based on that.

**Reviewer’s comment: Table Structure**: Uniformize format, including units, complete row numbering, WHO maximum values, and justify the absence of references in some rows.

**Authors’ response: **The authors are of the view that it will be clumsy to insert WHO limits for all the metals. Different articles investigated different metals; hence, the use of the word…exceeded or within the WHO limit as currently reported could suffice. On the aspect of units, the authors can confirm that units are indicated

**Reviewer’s comment: Legends and Abbreviations**: Include explanations for BDL, ND, and clarify terms such as "Si" and "exceeds limit" in the WHO limits column.

**Authors’ response: **That has been attended to

**Reviewer’s comment: Geographic Information**: Add a column in tables identifying the specific study region.

**Authors’ response: **The inclusion criteria for the study already indicated the region. The systematic review setting was the southwest region of Nigeria.

**Reviewer’s comment: Data Format**: Standardize the number of decimal places and metal nomenclature (acronyms or full names).

**Authors’ response: **That has been attended to

**Reviewer’s comment: Article Accessibility**: Identify the quantity of open access and subscription articles utilized.

**Authors’ response: **The authors had full access to all the included articles that were used.

**Reviewer’s comment: **It is suggested to provide quantitative summaries of contamination levels by food type and metal (ranges, medians).

**Authors’ response: **We can only report based on data provided by the authors. The different articles used different reporting formats. Since this was not an original research article, it will be difficult to harmonize and add that information consistently
